# Natural Hemp-Ginger Extract and Its Biological and Therapeutic Efficacy

**DOI:** 10.3390/molecules27227694

**Published:** 2022-11-09

**Authors:** Taja Žitek, Dragana Bjelić, Petra Kotnik, Andrej Golle, Staša Jurgec, Uroš Potočnik, Željko Knez, Matjaž Finšgar, Ivan Krajnc, Igor Krajnc, Maša Knez Marevci

**Affiliations:** 1 Laboratory for Separation Processes and Product Design, Faculty of Chemistry and Chemical Engineering, University of Maribor, Smetanova ul. 17, 2000 Maribor, Slovenia; 2 Laboratory for Analytical Chemistry and Industrial Analysis, Faculty of Chemistry and Chemical Engineering, University of Maribor, Smetanova ul. 17, 2000 Maribor, Slovenia; 3 Department of Chemistry, Faculty of Medicine, University of Maribor, Taborska ul. 8, 2000 Maribor, Slovenia; 4 National Laboratory for Health, Environment and Food, Prvomajska ul. 1, 2000 Maribor, Slovenia; 5 Center for Human Molecular Genetics and Pharmacogenomics, Faculty of Medicine, University of Maribor, Taborska ul. 8, 2000 Maribor, Slovenia; 6 Laboratory of Biochemistry, Molecular Biology and Genomics, Faculty of Chemistry and Chemical Engineering, University of Maribor, Smetanova ul. 17, 2000 Maribor, Slovenia; 7 Department of Internal Medicine, University of Maribor, Taborska ul. 8, 2000 Maribor, Slovenia; 8 Department of Cardiology and Angiology, University Clinical Center Maribor, Ljubljanska ul. 5, 2000 Maribor, Slovenia

**Keywords:** hemp, ginger, extract, skin diseases, WM-266-4

## Abstract

The prevention and treatment of skin diseases remains a major challenge in medicine. The search for natural active ingredients that can be used to prevent the development of the disease and complement treatment is on the rise. Natural extracts of ginger and hemp offer a wide range of bioactive compounds with potential health benefits. This study evaluates the effectiveness of hemp and ginger extract as a supportive treatment for skin diseases. It reports a synergistic effect of hemp and ginger extract. The contents of cannabinoids and components of ginger are determined, with the highest being CBD (587.17 ± 8.32 µg/g) and 6-gingerol (60.07 ± 0.40 µg/g). The minimum inhibitory concentration for *Staphylococcus aureus* (156.5 µg/mL), *Escherichia coli* (625.2 µg/mL) and *Candida albicans* (78.3 µg/mL) was also analyzed. Analysis of WM-266-4 cells revealed the greatest decrease in metabolic activity in cells exposed to the extract at a concentration of 1.00 µg/mL. Regarding the expression of genes associated with cellular processes, melanoma aggressiveness, resistance and cell survival, a significant difference was found in the expression of *ABCB5*, *CAV1* and *S100A9* compared with the control (cells not exposed to the extract).

## 1. Introduction

The incidence of skin diseases is increasing, which has enhanced the need for agents and therapeutics that alleviate symptoms or even cure disease. Skin diseases include psoriasis [1], actinic keratosis [2], rosacea [3], eczema [4], melanoma [5], basal cell carcinoma [6,7], squamous cell carcinoma [7] and many others. Such diseases are on the rise due to sun exposure, pollution and genetic susceptibility [8]. These diseases not only affect a person’s quality of life, but they are also dangerous from the point of view of the association with other diseases, such as cancer due to cardiac metastases. Specifically, 64% of patients with metastatic melanoma were also found to have cardiac metastases and to have a five-year survival rate of up to 20% [9,10]. Skin diseases can therefore escalate, significantly worsening the quality of life, shortening life expectancy and increasing the burden on the healthcare system [11]. On the other hand, some skin diseases, for example psoriasis, are also exacerbated by the presence of microorganisms such as *Staphylococcus aureus* [12], *Candida albicans* [12,13], *Streptococcus pyogenes* [14], *Staphylococcus epidermidis* [15], *Bacillus* spp., *Escherichia coli* [16] and *Enterococcus faecalis* [17].

Due to the short-term efficacy of therapeutic agents, possible side effects and their high cost, alternative natural treatment options, including active ingredients derived from natural extracts, are constantly sought. One example of a plant whose extract possesses therapeutic active ingredients is cannabis [18]. Cannabis extracts contain cannabinoids that inhibit keratinocyte proliferation and inflammation, thereby reducing the expression of keratins K6 and K16, which are typically upregulated in psoriasis [19]. Cannabinoids have great potential for the treatment of inflammatory skin diseases, as they can regulate cannabinoid receptor 1 and cannabinoid receptor 2 and reduce the activity of T-helper cells and keratinocytes [20,21]. Moreover, they not only have the effect of relieving the symptoms of inflammatory skin diseases, but they also have an anti-malignant effect in the case of melanoma and non-melanoma skin cancer [18,20,21]. Given that cannabinoids make up an insignificant portion of cannabis, decarboxylation is a key process for increasing the amount of cannabinoids, as it increases yield and facilitates the successful medical use of cannabis. Acids such as Δ9-tetrahydrocannabinolic acid A (THCA-A), cannabidiolic acid (CBDA), and cannabigerolic acid (CBGA) occur to a greater extent, and these are converted by the decarboxylation process to Δ9 tetrahydrocannabinol (Δ9-THC), cannabidiol (CBD) and cannabigerol (CBG), respectively [22,23,24]. Due to the pharmacological inactivity of CBDA, it is necessary to carry out a decarboxylation process induced by heat or light. Thus, at a temperature between 80 °C and 150 °C (in an exposure-time-dependent manner), CBDA is converted into a pharmacologically active CBD molecule, which is subsequently converted into cannabichromene (CBC) and cannabinol (CBN) in following steps [22,24].

In addition to cannabis, ginger also has many similar favourable properties. Ginger extract contains several biologically active compounds, which have analgesic and antibacterial properties and also increase the gastrointestinal tract’s motility [25]. In vivo experiments on mice have shown that ginger extract has antioxidant properties that alleviate damage to the gastric mucosa and promote healing [26,27]. Although further research is needed regarding ginger’s anti-inflammatory properties, ginger has an advantage over conventional non-steroidal anti-inflammatory drugs (NSAIDs), as it has minor gastrointestinal and renal side effects. Ginger components act as NSAIDs by inhibiting cyclooxygenase and lipoxygenase (pro-inflammatory enzymes), resulting in reduced biosynthesis of prostaglandins and leukotrienes [28]. In addition, numerous in vitro and in vivo animal studies have shown effective inhibition of carcinogenesis [29]. Not only is it effective in vitro and in vivo in animals, but it has been shown to be an effective adjuvant in the treatment of cancer with chemotherapy, as it reduces the levels of oxidative markers and the severity of acute chemotherapy-induced nausea and adult cancer patients [30,31]. Other effects of ginger extract include inhibition of carcinogenesis and anti-cancerous effects for cancers such as ovarian, breast, gastric, colorectal, pancreatic and skin cancers, which results from bioactive components of the extract such as 6-gingerol and 6-shogaol [32,33,34].

Given that both hemp extract and ginger extract have been found to be effective in the treatment of various skin diseases, it is possible to speculate about their positive synergistic effect. In our previous work [35], the extract with the highest antioxidant activity was determined by comparing different ways of preparing the input material and extraction methods. The extract from the previous research achieved the best results in the inhibition of microbes and the metabolic activity of WM-266-4 melanoma cells. In the current study, the optimized recovery of the extract is described, and its content is investigated by LC-MS/MS analysis. The metabolic activity of WM-266-4 cells was also analyzed in the presence of several concentrations of the extract (0.01–30.00 mg/mL), and the expression of the genes caveolin 1 (*CAV1*), ATP binding cassette subfamily B member 5 (*ABCB5*), S100 calcium-binding protein A9 (*S100A9*) and sirtuin 1 (*SIRT1*) was measured at the mRNA level. Moreover, the minimum inhibitory concentration for *Staphylococcus aureus*, *Escherichia coli* and *Candida albicans* was also determined.

## 2. Results

### 2.1. Extract Characteristics

The contents of the components of the hemp-ginger extract are summarized in Table 1 and Table 2. Table 1 shows the amount of the selected cannabinoids in mg per g of extract. Because the aim was to achieve the highest possible content of the CBD component, the extract was decarboxylated at 140 °C for 15 min. During the decarboxylation process, the acid forms of cannabinoids were converted into active forms (based on the results obtained, which are presented in Table 1). The content of the acid form such as CBDA (37.90 mg/g) in the extract is significantly lower than the content of the active form, CBD, which represents 587.17 mg/g of the extract.

### 2.2. Minimum Inhibitory Concentration for Microorganisms

Antimicrobial potential (using *Staphylococcus aureus*, *Escherichia coli* and *Candida albicans*) was tested for the extract. The minimum inhibitory concentrations (MIC) of extract required to inhibit microbial growth were: 78.3 µg/mL for inhibition of the fungus *C. albicans*, 156.5 µg/mL for inhibition of the Gram-positive bacterium *S. aureus* and 625.2 µg/mL for inhibition of the Gram-negative bacterium *E. coli*.

### 2.3. Metabolic Activity of WM-266-4 Cells and Appoptosis

**Figure 1:**
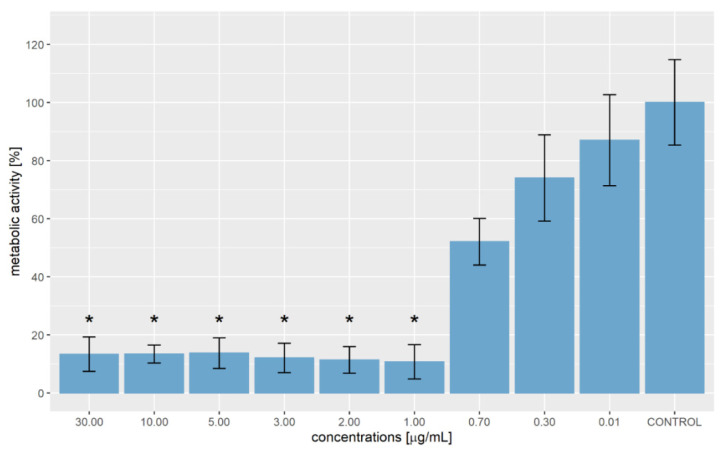
Metabolic activity of WM-266-4 cells exposed to different concentrations of hemp-ginger extract. Data generated in five replicates in each experiment for three independent experiments are shown as mean ± SD. Statistical significance, indicated with *, was defined as *p* < 0.050 compared with the control group (Kruskal-Wallis post hoc Dunn test).

**Figure 2:**
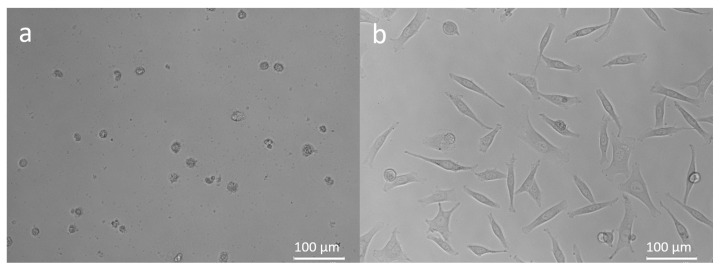
Morphology of WM-266-4 cells exposed to 1.00 µg/mL hemp-ginger extract (**a**) and control group: WM-266-4 cells in growth medium (**b**). Magnification 200×.

The combination of hemp and ginger extract has a promising inhibitory effect on melanoma cells [35]. Figure 1 shows the metabolic activity of WM-266-4 cells in the absence of extract and in the presence of extract at various concentrations (0.01–30.00 µg/mL). Treatment of WM-266-4 melanoma cells with hemp-ginger extract resulted in a significant decrease in metabolic activity in the presence of extract concentrations of 1.00 µg/mL and higher compared with the metabolic activity of melanoma cells in a medium without extract (control). The data were non-normally distributed and Levene’s test indicated equal variances (F = 0.995, *p* = 0.474). Therefore, the Kruskal-Wallis test was performed, which indicated a statistically significant difference in mean metabolic activity across concentrations (χ^2^ = 24.823, *p* = 0.003). The post hoc Dunn test confirmed significant differences between the mean metabolic activity of the control group (without extract) and the mean metabolic activity of the cells treated with extract of concentration 1.00 µg/mL and higher, as shown in Figure 1. The latter is also shown by the morphology of the cells in Figure 2. The images show the shape of the cells after application of the extract (1.00 µg/mL) (Figure 2b) and the shape of the cells in the control group (Figure 2a). Irregular cell shapes can be seen, indicating an apoptotic response.

The test also confirmed significant differences in mean metabolic activities of cells exposed to various concentrations. Table 3 shows the results of the Kruskal-Wallis post hoc Dunn test at the significance level of *p* < 0.050 (shaded with grey).

A statistically significant difference in mean metabolic activity was found when comparing control cells with cells exposed to extract concentrations of 1.00 µg/mL and higher. Moreover, a statistically significant difference in mean metabolic activity was found in cells treated with an extract concentration of 0.01 µg/mL in comparison with cells treated with 1.00 µg/mL and higher extract concentrations. Despite the increase in extract concentration to 0.30 µg/mL, mean metabolic activity was still significantly different from the mean metabolic activity of cells treated with extract concentrations of 1.00 µg/mL, 2.00 µg/mL and 3.00 µg/mL. In the presence of 0.70 µg/mL of extract, the average metabolic activity dropped to 52.00 ± 8.04%, but it was still significantly higher compared to the average metabolic activity of cells in the presence of an extract concentration of 1.00 µg/mL, where the metabolic activity was 10.71 ± 5.89%.

For comparison, the metabolic activity of epidermal melanocytes of the skin was measured when the same concentrations were applied, and it was found that there was no visible change in the metabolic activity of HEM cells when the extract was applied in the concentration range compared to the control. At higher concentrations, the metabolic activity of HEM cells changed significantly. Therefore, we concluded that the optimal application of the extract to the cells is up to concentrations of 2 µg/mL. However, in the apoptosis assay, we found the optimal dose to be up to 1 µg/mL, as we detected a 50% apoptotic response at a concentration of 2 µg/mL. 

**Figure 3:**
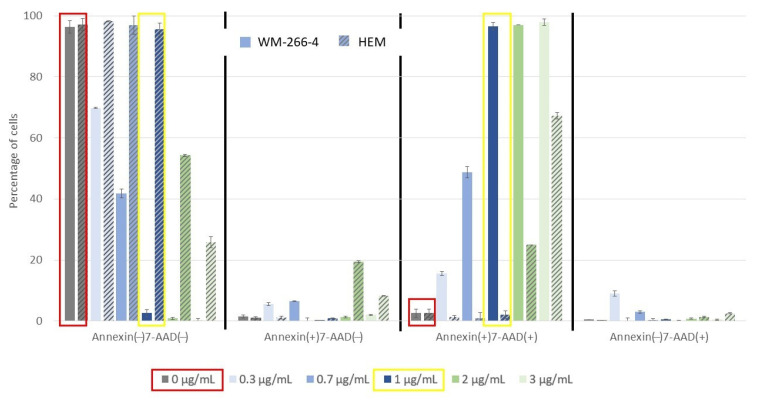
Apoptotic activity of extract against melanoma WM-266-4 and HEM cells at various extract concentrations (0 µg/mL, 0.3 µg/mL, 0.7 µg/mL, 1 µg/mL, 2 µg/mL and 3 µg/mL).

Therefore, when investigating the extent of apoptotic cell death, WM-266-4 cells and HEM cells were treated with the extract at various concentrations (0–3 µg/mL), and the cells were stained with Muse™ Annexin V & Dead Cell Reagent and recorded using Muse™ Cell Analyzer. Representative results of the assay with untreated WM-266-4 cells and HEM cells are shown in Figure 3. The graph shows the percentage of live, early apoptotic, late apoptotic and cellular debris represented by Annexin(−)7-AAD(−), Annexin(+)7-AAD(−), Annexin(+)7-AAD(+) and Annexin(−)7-AAD(+), respectively.

### 2.4. Molecular Analysis

In accordance with the results of the analysis of the metabolic activity of cells exposed to different concentrations of extracts, groups with the most statistically significant difference were selected, and gene expression analysis was performed. Selected groups compared with the control cells were exposed to an extract concentration of 1.00 µg/mL, 0.30 µg/mL and 0.01 µg/mL. The expression of reference genes (*B2M*, *GAPDH*) proved to be stable and without statistically significant differences (χ^2^ = 4.918, *p* = 0.178).

**Figure 4:**
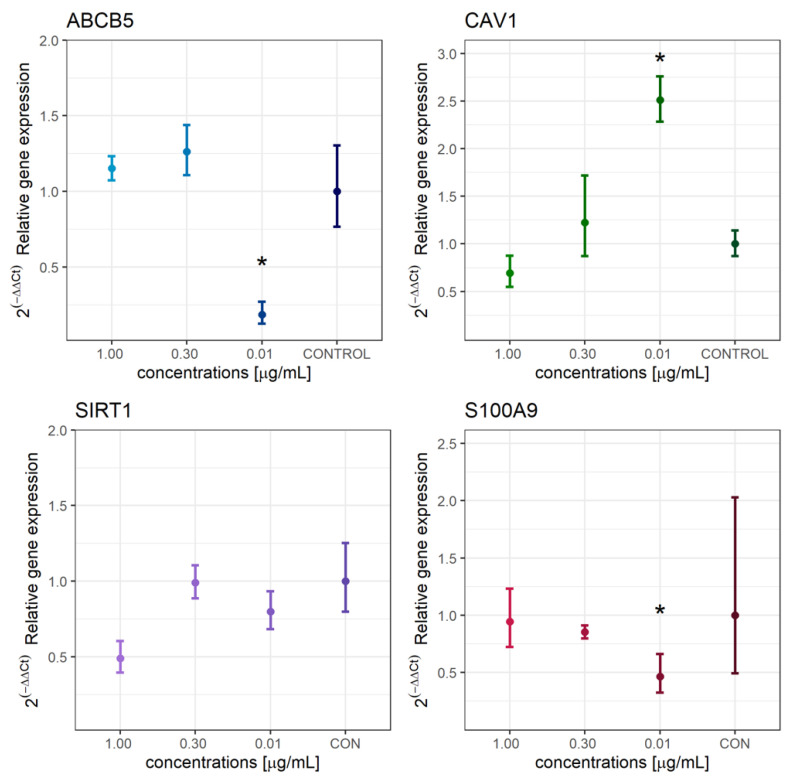
Analysis of relative expression of target genes *ABCB5*, *CAV1*, *SIRT1* and *S100A9* in WM-266-4 cells exposed to different concentrations of extract (1.00 µg/mL, 0.30 µg/mL and 0.01 µg/mL) and in control WM-266-4 cells (no extract present). Data from 3 independent experiments are presented as 2^−ΔΔCt^ expressions relative to the control (monolayer culturing)  ±  standard error of expression. The statistical difference compared to the control was determined using the Kruskal-Wallis post hoc Dunn test (* *p* < 0.050).

Relative expression of *ABCB5*, *CAV1*, *SIRT1* and *S100A9* is shown in Figure 4. Compared to the control, extract concentrations of 1.00 µg/mL and 0.30 µg/mL had a minor effect on the increase of *ABCB5* expression (1.15-fold and 1.26-fold, respectively). In contrast, a concentration of 0.01 µg/mL induced downregulation of *ABCB5* (0.19-fold), which was significantly lower than the control (*p* = 0.029). The results of our research show that even a low concentration, more precisely 0.01 µg/mL, is sufficient for significantly reduced expression. *ABCB5*, which is also associated with therapeutic resistance, provides a promising therapeutic target for melanoma [36]. Although higher concentrations did not induce a decrease in *ABCB5* expression, metabolic activity was significantly lower compared to the control. On the other hand, at a concentration of 0.01 µg/mL, a significantly increased expression of *CAV1* was observed compared to control (*p* = 0.016). The change in expression was 2.51-fold. Expression also changed as the extract concentration increased to 0.30 µg/mL and 1.00 µg/mL, with slight upregulation (1.23-fold) and slight downregulation (0.69-fold) compared to the control, respectively. In the case of *SIRT1*, downregulation was observed at low concentrations of extract; at the lowest concentration, the expression was 0.68-fold of the control, and at the concentration of 0.10 µg/mL, the expression was 0.88-fold of the control. The largest difference in *SIRT1* expression was found at the highest extract concentration, as downregulation (0.49-fold of control) was observed. However, the changes were not statistically significant. *S1AA09* expression decreased with decreasing extract concentration. The extract concentration of 0.01 µg/mL was sufficient for a significant downregulation to 0.46-fold of the control (*p* = 0.024). At a concentration of 0.3 µg/mL, expression decreased to 0.85-fold of the control, and at concentration 1.0 µg/mL, there was a minor decrease to 0.94-fold of the control.

## 3. Discussion

The minimal inhibitory concentration (MIC) value was determined for three microorganisms (*Staphylococcus aureus, Escherichia coli* and *Candida albicans*). In this study, the antimicrobial potential for both the bacteria and fungus were confirmed. The highest value was determined for *E. coli* (MIC = 625.2 µg/mL). Farha and colleagues studied the effects of several cannabinoid analogues against *E. coli* [37], but none showed bactericidal activity against *E. coli* with consistent MIC values > 128 μg/mL. This is consistent with the present study where the MIC value was found to be 625.2 μg/mL. On the other hand, the MIC values in this study for *S. aureus* and *C. albicans* were 156.5 μg/mL and 78.3 μg/mL, respectively. The antimicrobial potential of the extract is probably due to the cannabinoids present. A study by van Klingeren and Ham reported that both components ∆9-THC and CBD were active against several *S. aureous* isolates, with MIC of 1–5 μg/mL [38]. Turner et al. evaluated the antibacterial and antifungal properties of the cannabichrome analogue plate [39]. Appendino et al. investigated the antibacterial activity of five major cannabinoids against *S. aureus* [40]. Cannabidiol acid (CBDA) was found to have high antimicrobial activity (MIC = 2 μg/mL). Martinenghi et al. reported that CBD has MIC values in the range of 1–2 μg/mL vs. CBDA (2–4 μg/mL) for several Gram-positive pathogens [41]. Previous studies on active inhibition of microorganisms by alcoholic cannabis extract for all three microorganisms [40]. Cannabinoids, which are the main constituents of hemp extract, have also been reported to have anti-cancerous activity [35,42,43]. 

Cannabidiol (CBD) is a bioactive molecule that successfully inhibits the growth of melanoma cells (in this study, the extract contained 587 mg/g of CBD). A CBD oil concentration of 0.04 mg/mL effectively inhibited the growth of B16 mouse melanoma cells in vitro [44]. Another study reported a positive effect of hemp extract as supportive therapy in radiation. It found an antitumor effect and an inhibitory effect on metabolic activity of B16F10 mouse melanoma cells, which are radioresistant [45]. The inhibitory effect on metabolic activity and cell growth was observed not only in melanoma cells but also in NIH3T3 fibroblasts, A549 lung cancer cells, MDA-MB-231 breast cancer cells, Lenz kidney cells and SNU-C4 colon cancer cells. The concentration of CBD required for the inhibitory effect on cancer cells ranges from 5 μM to 80 μM [46]. Ginger extract also has an inhibitory effect on tumor cells and affects the metabolic activity of cancer cells [47]. Moreover, it has antibacterial properties, with a minimum inhibition concentration (MIC) of 1.0 mg/mL and a minimum bactericide concentration (MBC) of 2.0 mg/mL for Staphylococcus aureus. For *E. coli* the MIC is 2.0 mg/mL, and the MBC values are 4.0 mg/mL [48]. Therefore, the combination of hemp and ginger seems promising. The present study showed that the metabolic activity of WM-266-4 cells decreased to 52.00 ± 8.04% at an extract concentration of 0.70 µg/mL, while the metabolic activity dropped to 10.71 ± 5.89% at a concentration of 1.00 µg/mL. As the concentration increases, the metabolic activity of the cells remains at approximately 13%. The cause of a small and insignificant change in the metabolic activity of cells exposed to hemp-ginger extract concentrations between 1.00 µg/mL and 30.00 µg/mL is most likely the cells that are the most durable, and the extract does not have such an effect on their metabolic activity. Given that the cells were exposed to the extract for 24 h, we can conclude that prolonging the time could also reduce the metabolic activity of even more durable cells.

To inhibit the activity of melanoma cells and intracellular processes, it is crucial for therapeutic agents to influence the expression of genes responsible for cancer aggressiveness, the development of metastases and the inflammatory response in other skin diseases. One of the genes whose increased expression is linked to greater cancer aggressiveness and drug resistance is *ABCB5* [36,49]. Given that *ABCB5* plays a key role in melanoma growth and the promotion of melanoma metastases, silencing this gene is crucial in inhibiting metastasis development [50,51]. The present study shows that a minimum concentration (0.01 µg/mL) is sufficient for significant downregulation of *ABCB5*. Therefore, hemp–ginger extract reduces the expression of this gene, which suggest that it could reduce the aggressiveness of melanoma and consequently also help reduce resistance to chemotherapy. From the obtained results, it can be concluded that, even at low concentrations, it is possible to reduce the aggressiveness of the melanoma. On the other hand, *ABCB5* expression increased at higher concentrations, i.e., 0.30 µg/mL and 1.00 µg/mL. From significantly lower metabolic activity and increased expression, it can be assumed that the surviving cells were much more durable and that this consequently increased the expression slightly.

Compared with *ABCB5*, the role of *CAV1* in skin diseases is more complex, as expression depends on the type of melanoma. Metastatic cutaneous, primary cutaneous and primary uveal cells were used for quantitative RT-PCR and Western blotting, where significantly higher *CAV1* expression was found in cutaneous cells compared with uveal cells [52]. *CAV1* can act as a tumor suppressor or tumor promoter depending on the cell type, although its role in melanoma has not yet been elucidated [53]. *CAV1* expression has been shown to stimulate the proliferation of B16F10 melanoma cells while inhibiting migration and invasion in vitro [54]. Although increased expression of *CAV1* in tumor cells is thought to be associated with aggressiveness and poor survival [55], the correlation between *CAV1* expression and melanoma remains largely unexplored. Our study showed that *CAV1* expression increased significantly (2.51-fold) in the presence of the extract at a concentration of 0.01 µg/mL, while metabolic activity decreased to 86.95 ± 15.68%. On the other hand, in the presence of the extract with a concentration of 1.00 µg/mL, *CAV1* expression decreased (0.69-fold) compared with the control, while metabolic activity fell to 10.71 ± 5.89%. Thus, if a decrease in melanoma aggressiveness and metastasis development is associated with a decrease in *CAV1* expression, a higher extract concentration is required. One possible explanation for why the expression increased at low extract concentrations is the response of the cells and their struggle for survival. For a more substantiated explanation, it is necessary to investigate the relationship of *CAV1* with the aggressiveness of melanoma and the development of metastases in more detail in the future.

*SIRT1* encodes the SIRT1 protein, a member of sirtuin family that is present in both the nucleus and cytoplasm depending on the cell type [56]. It plays a crucial role in the process of cellular regulation, as it deacetylates histones and non-histone proteins. It also acts as a metabolic regulator [57,58]. SIRT1 is involved in tumor initiation, promoting cell proliferation and drug resistance, and it is overexpressed in human melanoma [59]. Therefore, it represents one of the possible target genes with which we could reduce the aggressiveness of melanoma by silencing its expression [60]. In general, in the presence of hemp–ginger extract, expression of *SIRT1* was downregulated, suggesting that *SIRT1* could act as a silencer. However, no statistical significance was found.

*S100A9* encodes the protein of the same name, which plays an important role in the regulation of a number of cellular processes. Together with S100A8, it forms a heterodimer called calprotectin, which can serve as a biomarker for inflammatory bowel diseases [61]. It can also serve as a biomarker for melanoma, as overexpression of this gene has been found melanoma cells compared with healthy cells [62]. Therefore, silencing this gene could disrupt normal cellular processes in melanoma cells. A significant decrease in *S100A9* expression was observed at an extract concentration of 0.01 µg/mL, and as the concentration increased, the expression approached that of the control group. Given that the metabolic activity of the cells is also significantly reduced at the highest concentration of the extract (1.00 µg/mL), the approximation of the expression to the control group is very likely due to the survival of more durable cells.

Considering that the studied extract mixture contained higher amounts of CBD and 6-gingerol, it can be concluded that these two components influence the decrease in metabolic activity and the observed difference in gene expression compared to the control. In an in vivo study using mice, 6-gingerol was found to have chemopreventive properties, as it reduced the volume of skin tumors and prevented tumor formation [63]. In ginger extract, 6-gingerol and 6-shogaol contribute the most to chemopreventive efficacy [64]. As for CBD, it was found to inhibit melanoma cells in vitro, specifically in B16 mice melanoma cells [44]. It has also been shown to maintain keratinocyte proteostasis and prevent UVA/UVB-induced metabolic changes in UVA- and UVB-irradiated keratinocytes [65]. CBD is known for its potent antiproliferative and pro-apoptotic effects in a variety of cancers [66]. On the other hand, the presence of a wide range of active compounds in the extract may produce a synergistic effect [67,68].

## 4. Materials and Methods

### 4.1. Materials

Dried industrial hemp (leaves, flowers (buds) and stems) of the species Kc Dora was purchased from local growers in Slovenia (Makoter farm, Cven, Slovenia) and then mechanically processed to obtain six fractions, which was described in detail in our previous studies. The fraction with the most cannabinoids was used for extraction. Coarsely ground ginger was purchased from Alfred Galke GmbH (Samtgemeinde Bad Grund, Germany). The average particle size of both materials was ground to 0.1 mm.

### 4.2. Process and Preparation of Extract

**Figure 5:**
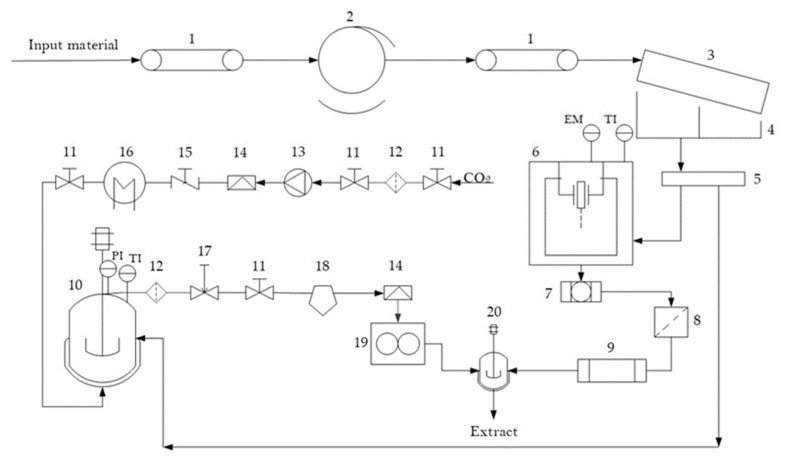
Simplified scheme of the process: 1: conveyor, chain, closed; 2: rotary drum; 3: sowing disc with a slope; 4: collection container; 5: bowl with stirrer; 6: ultrasonic vessel, 7: filter one; 8: filter two; 9: rotavapour; 10: autoclave; 11: valve; 12: HP filter; 13: HP pump; 14: rupture disc; 15: one-way valve; 16: heat exchanger; 17: regulating valve; 18: separator; 19: flowmeter; 20: mixer.

The procedure for the preparation and extraction of hemp and ginger is shown in Figure 5. This procedure was explained in detail in a previous study [35,69]. The novelty of our study is the use of raw dried material that is previously mechanically treated for better selectivity (higher cannabinoid content) and then mixed with ginger to achieve better bioavailability of the extract.

The extraction process consisted of a conveyor belt through which a rotating drum of dried industrial hemp was passed. In the drum, the stems and seeds were separated from the flowers and leaves. The leaves and flowers were further sieved onto a 100-micron inclined sieve. Below the sieve was a collecting bin divided into two parts. The material collected in the first part was used for the extraction process, which consists of ultrasonic extraction (UE) and supercritical fluid extraction (SFE). Briefly, hemp and ginger were finely ground in a weight ratio 1:1. Because antimicrobial activity and high contents of CBD, antioxidants and total phenols were found in SFE and UE in our previous work, the same extraction procedure was carried out in this work. Briefly, ethanol was used as a solvent for UE performance, and CO_2_ was used as a solvent for supercritical fluid in SFE. The SFE of mixed material was performed in the SFE system at a temperature of 60 °C and a pressure of 300 bar. The SFE experiments were conducted by the solvent-to-feed (S/F) ratio of 8.143. The procedure for the SFE is shown in Figure 5 (numbers 10–19). It consisted of high-pressure filters, a high-pressure pump, rupture discs, a heat exchanger, an autoclave with a heat jacket system, a flowmeter, corresponding pipes and valves. The gas was introduced through the pipes with the compressor. The supercritical extract was collected at the bottom of the separator and removed into a mixer (number 20 in Figure 5), where it was mixed with the ultrasonic extract. The yield of the supercritical fluid extract was 14.72 ± 0.32%. The yield of the ultrasonic extract was 26.76 ± 1.19%. The feeding of both extracts into the separator was controlled so that they were in a 1:1 ratio. As mentioned above, SFE- and UE-obtained extracts showed antimicrobial effects and high content of CBD, antioxidants and total phenols in our previous work; therefore, in this work, SFE and UE extracts were mixed in a weight ratio of 1:1.

### 4.3. Liquid Chromatography-Tandem Mass Spectrometry Analyses

The content of cannabinoids and ginger constituents was determined by liquid chromatography coupled with tandem mass spectrometry (LC-MS/MS) using an Agilent 1200 HPLC instrument (Agilent Technologies, Santa Clara, CA, USA) with an Agilent 6460 Triple Quad JetStream mass detector (QqQ, Agilent Technologies, Santa Clara, CA, USA). A chromatographic precolumn Agilent Poroshell EC-C18 (4.6 μm particles) and an Agilent Poroshell EC-C18 column (2.7 μm particles, 100 × 2.1 mm ID) were used to separate cannabinoids. Ginger constituents were separated using the Phenomenex Kinetex C18 column (2.6 μm particles, 150 × 2.1 mm ID). For the determination of cannabinoids and ginger components, the mobile phase consisted of 0.1% formic acid in ultrapure water (mobile phase A) and 0.1% formic acid in acetonitrile (mobile phase B). The separation of the components was carried out by gradient method: 0 min 34% B, 8 min 34% B, 12 min 95% B, 13 min 95% B, 14 min 34% B and 20 min 34%. The flowrate through the column was 0.2 mL/min. The column temperature was 35 °C. For the determination of ginger components, the optimized gradient program of the mobile phases was as follows: 0 min 50% B, 3 min 90% B, 6 min 90% B and 7 min 50% B, which was held for 5 min. The flow rate of the mobile phase was 0.5 mL/min, and the temperature of the column was 30 °C. The multiple reaction monitoring mode for individual ion transition was used for component identification and quantification.

### 4.4. Microdilution Method for Determination of Antimicrobial Activity

The microdilution method was performed using cation-adjusted Mueller-Hinton broth (MH). The MH broth was supplemented with lysed horse blood and β-NAD (MH-F broth). The antimicrobial potential of *Staphylococcus aureus* (MH), *Escherichia coli* (ATCC 25,922, ATCC, Wesel, Germany) and *Candida albicans* (ATCC 60,193, ATCC, Wesel, Germany) were determined using the method as presented previously [35]. The emulsion of the extract was prepared in eight different concentrations (195–12,500 µg/mL). The inoculum concentration was 10^8^ CFU/mL. The process of emulsification was prepared with water-based MH and oil-based hemp-ginger extract. Emulsification was made with Tween 80 (T80) emulsifying agent at room temperature with a rotor-stator homogenizer (Homogenizer, Polytron Pt1200, Kinematica AG, Luzern, Switzerland). The extract suspension in MH was homogenized at 70,000 g. The microdilution procedure was evaluated by the pentaplicates repeatability procedure and by the blue resazurin colour that indicated the presence of bacteria. It was also evaluated with the negative and positive controls.

### 4.5. Metabolic Activity of WM-266-4 and Normal Human Epidermal Melanocytes

**Figure 6:**
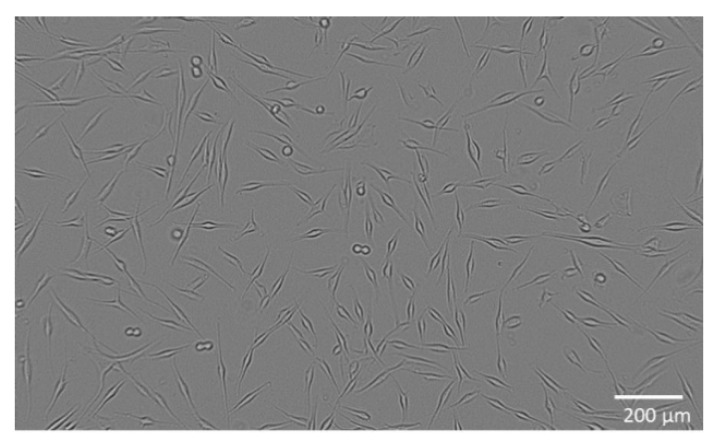
Melanoma cells WM-266-4 during cultivation.

**Figure 7:**
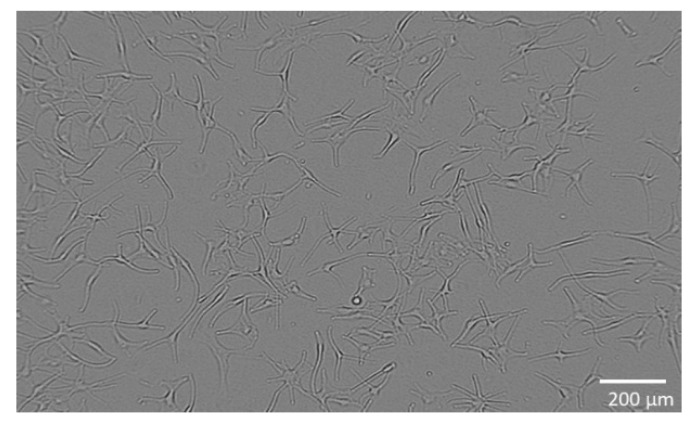
Normal human epidermal melanocytes during cultivation.

The skin metastatic melanoma cell line WM-266-4 (ATCC^®^ CRL1676™, Manassas, VA, USA) was purchased from American Type Culture Collection (ATCC, Manassas, VA, USA) and cultured in Eagle’s Minimum Essential Medium (EMEM, ATCC^®^ 30-2003™, Manassas, VA, USA) containing 10 vol.% fetal bovine serum (FBS, ATCC^®^ 30-2021™, Manassas, VA, USA) with addition of 0.02 vol.% MycoZap™ Plus-CL (Lonza, Portsmouth, NH, USA). The cells were incubated at 37 °C, 5% CO_2_, ≥90% RH and plated at a density of 2 × 10^4^ viable cells per well in 96-well culture plates. The cells were cultured in a complete medium for 24 h to allow cell attachment (Figure 6). Normal human epidermal melanocytes (NHEM) (SI-104-05A, Taufkirchen, Germany) are primary cells and were grown in a complete medium: melanocyte growth medium (SI-135-500, Manassas, VA, USA). The cells were plated at a density of 1 × 10^4^ viable cells per well in 96-well culture plates and cultured for 24 h in medium to allow cell attachment (Figure 7).

To analyse the metabolic activity of the cells, they were exposed to different concentrations of the extract (0.01–30.00 µg/mL) and incubated for 24 h, with five replicates performed. Control cells were not exposed to the extract but were cultured in the medium for the same time and under the same conditions. For cells’ metabolic activity measurement, a WST 8 Colorimetric Cell Viability Kit I (PromoKine, PromoCell, Heidelberg, Germany, EU) was used according to the manufacturer’s instructions. Absorbance (*A*) was measured spectrophotometrically at 570 nm (*A*_570_) (background *A* was measured at 630 nm (*A*_630_)) in pentaplicates for all samples. The percentage of the cells’ metabolic activity (*MA*) was calculated with the following equation:

$$MA=\left(\frac{\left(A_{570}-A_{630}\right)\;test\;sample\;value}{\left(A_{570}-A_{630}\right)\;control\;sample\;value}\right)\times 100$$

where *A* represents the average value of absorbance calculated from five replicates. Additionally, cell morphology was observed with an inverted microscope (DM16000B, Leica) using a digital camera (DFC365 FX Leica, Lake City, IL, USA).

### 4.6. Determination of Cell Apoptosis

The level of advancement of the apoptosis process was determined using a Muse Cell Analyzer and Muse Annexin V and Dead Cell Kit (Luminex, Commercial Ave, Northbrook, IL, USA) described in detail in our previous work [69]. Briefly, after each experiment, cells were trypsinized, and 100 μL of cell suspension was prepared for analysis. Next, 100 μL of Annexin V and dead cell reagent was added to each sample and mixed. After 20 min in the dark, it was analyzed with Muse Cell Analyzer. Each experiment was performed in pentaplicate, and the mean value was determined.

### 4.7. Molecular Analysis-Gene Expression

For analysis of gene expression, cells were grown under the same conditions as described in Section 4.5 (Determination of the Cells’ Metabolic Activity). RNA was extracted from the melanoma cell line WM 266-4 using RNeasy^®^ Mini Kit (Qiagen, Hilden, Germany) according to the manufacturer’s protocol. Briefly, cells were lysed and homogenized in Buffer RTL containing β-mercaptoethanol (10 µL β-mercaptoethanol per 1 mL of Buffer RTL). Ethanol was added, and samples were transferred to RNeasy spin column and placed in a 2 mL collection tube. RNeasy spin columns with samples were washed with Buffer RW1 and twice with Buffer RPE. RNase-free water was added directly to the RNeasy spin column membrane to rinse RNA into a new collection tube. RNA quality was assessed using a Synergy 2 spectrophotometer (Biotek, Winooski, VT, USA). Samples that did not contain sufficient RNA concentration required for cDNA transcription and further analysis were excluded. In total, 100 ng of RNA from each sample was reverse transcribed into cDNA using a High-Capacity cDNA Reverse Transcription Kit (Thermo Fisher, Waltham, MA, USA). Primers for gene expression of target genes (*ABCB5*, *CAV1*, *SIRT1*, *S100A9*) as well as reference genes (*B2M*, *GAPDH*) were designed using Integrated DNA Technologies OligoAnalyzer 3.1 (IDT; Coralville, IA, USA). Gene expression was measured using QuantStudio 12K Flex Real-Time PCR System (Applied Biosystems, Singapore, Singapore). The PCR cycling conditions were as follows: initial denaturation at 95 °C for 5 min, followed by 55 cycles of 95 °C for 10 s, 60 °C for 15 s and 72 °C for 15 s. The relative expression of the target genes was normalized using geometric means of *B2M* and *GAPDH* reference genes, and quantification was performed using the comparative Ct (2^−ΔΔCt^) method, as described by Livak and Schmittgen [70].

Table 4 summarizes the primer sequences with the corresponding mRNA sequences and the corresponding National Center for Biotechnology Information Nucleotide database [71] accession numbers of transcription variants, the sequences of which are covered by these primers.

### 4.8. Statistical Analysis

Dixon’s and Grubbs’ statistical tests were used to detect possible outliers of the results obtained from the pentaplicate measurements of five replicates of cells’ metabolic activity performed for each extract and control sample concentration. All numerical data are presented as mean ± standard deviation (SD) from three independent experiments unless otherwise stated. First, data were analyzed using Shapiro-Wilk normality test. Secondly, Levene’s test for homogeneity of variances was performed. Data with normal distribution were further analyzed by an ANOVA test followed by post hoc analysis using the Dunnett’s test or Welch’s test post hoc Dunnett’s T3-test. However, data with non-normal distribution were analyzed using the Kruskal-Wallis H-test followed by post hoc analysis using the Dunn test. The stability of reference genes was assessed using Kruskal-Wallis H-test and using 2^−ΔCt^ calculation of raw data. Expression data were analyzed using 2^−ΔΔCt^ calculation, where *p* < 0.050 was considered statistically significant. Analyses were performed using R software version 4.0.3 (The R Foundation for Statistical Computing, Vienna, Austria) [72] and RStudio Version 1.3.1093 (RStudio, Inc., Boston, MA, USA) supported by the following packages: outliers, ggplot2, and lawstat.

## 5. Conclusions

This study demonstrates high cannabinoid contents in the extract using two methods of extract preparation (supercritical fluid extraction with CO_2_ and ethanolic ultrasonic extraction). A study on the combination of components demonstrated antimicrobial and anti-cancer potential. Analysis of the metabolic activities of the cells as a function of the concentrations of the extract to which they were exposed for 24 h showed that a concentration of 1.00 µg/mL is required for a significant decrease in metabolic activity. A more detailed insight into intracellular events was provided by the analysis of gene expression using qRT-PCR, which revealed that an extract concentration of 0.01 µg/mL was sufficient to induce significant changes in the expression of *ABCB5*, *CAV1* and *S100A9*.

## Figures and Tables

**Table 1:** Content of cannabinoids in hemp-ginger extract.

Cannabinoid	CBC	CBD	CBDA	CBGA	CBN	THC	THCA
[mg/g]	9.50 ± 0.42	587.17 ± 8.32	37.90 ± 0.40	11.12 ± 0.03	1.54 ± 0.01	14.73 ± 0.40	ND

**Table 2:** Content of different ginger components in hemp-ginger extract.

Component	6-Gingerol	6-Shogaol	8-Gingerol	8-Shogaol	10-Gingerol	10-Shogaol
[mg/g]	60.07 ± 0.40	27.88 ± 0.08	10.48 ± 0.04	2.98 ± 0.01	7.66 ± 0.02	4.89 ± 0.01

**Table 3:** Kruskal-Wallis post hoc Dunn test for comparison of mean metabolic activities in the presence of different concentrations of extract. The *p* values are reported in the table.

*c* [µg/mL]	30.00	10.00	5.00	3.00	2.00	1.00	0.70	0.30	0.01
*10.00*	0.463								
*5.00*	0.472	0.491							
*3.00*	0.289	0.258	0.266						
*2.00*	0.243	0.215	0.222	0.445					
*1.00*	0.159	0.138	0.143	0.330	0.382				
*0.70*	0.133	0.154	0.148	0.047	0.035	0.017			
*0.30*	0.063	0.075	0.072	0.018	0.013	0.006	0.338		
*0.01*	0.026	0.032	0.030	0.006	0.004	0.002	0.202	0.338	
*CON*	0.009	0.012	0.011	0.002	0.001	0.000	0.105	0.202	0.338

**Table 4:** Primer sequences, accession numbers and amplicon length of target genes and reference genes.

Gene	Accession Number	Forward (F) and Reverse (R) Primers	Amplicon Length [bp]
*ABCB5*	NM_001163993.3	F: TTGAAACCTTCGCAATAGCC	224
		R: GACCAAGGCGACTGTCTCT	
*CAV1*	NM_001753.5	F: GCAACTCGCTTTAGGTCAGC	201
		R: TCAGCCCTATTGGTCCACTT	
*SIRT1*	NM_012238.5	F: TGGAACAGGTTGCGGGAATC	106
		R: CCTCGTACAGCTTCACAGTCA	
*S100A9*	NM_002965.4	F: CATGGAGGACCTGGACACAAA	171
		R: CCACTGTGGTCTTAGGGGGT	
*B2M*	NM_004048.2	F: TTCTGGCCTGGAGGCTATC	86
		R: TCAGGAAATTTGACTTTCCATTC	
*GAPDH*	NM_002046.7	F: GAAGGTGAAGGTCGGAGTC	226
		R: GAAGATGGTGATGGGATTTC	

## Data Availability

The original contributions presented in the study are included in the article; further inquiries can be directed to the corresponding authors.

## Sample Availability

Samples of the compounds are not available from the authors.
